# White-Matter Connectivity and General Movements in Infants with Perinatal Brain Injury

**DOI:** 10.3390/brainsci15040341

**Published:** 2025-03-26

**Authors:** Ellen N. Sutter, Jose Guerrero-Gonzalez, Cameron P. Casey, Douglas C. Dean, Andrea de Abreu e Gouvea, Colleen Peyton, Ryan M. McAdams, Bernadette T. Gillick

**Affiliations:** 1 Department of Family Medicine and Community Health, University of Minnesota, 420 Delaware St. SE, Minneapolis, MN 55455, USA; esutter@bu.edu; 2 Waisman Center, University of Wisconsin-Madison, 1500 Highland Ave., Madison, WI 53705, USA; 3 Department of Pediatrics, University of Wisconsin-Madison, 600 Highland Ave., Madison, WI 53705, USA; 4 Department of Medical Physics, University of Wisconsin-Madison, 1111 Highland Ave. #1005, Madison, WI 53705, USA; 5 Department of Physical Therapy and Human Movement Sciences, Northwestern University, 645 North Michigan Ave. Suite 1100, Chicago, IL 60611, USA; 6 Department of Pediatrics, Northwestern University, 225 E. Chicago Ave., Chicago, IL 60611, USA

**Keywords:** cerebral palsy, magnetic resonance imaging, diffusion tensor imaging, perinatal brain injury, general movements assessment

## Abstract

Background/Objectives: Cerebral palsy (CP), often caused by early brain injury such as perinatal stroke or hemorrhage, is the most common lifelong motor disability. Early identification of at-risk infants and timely access to rehabilitation interventions are essential for improving long-term outcomes. The General Movements Assessment (GMA), performed in the first months of life, has high sensitivity and specificity to predict CP; however, the neurological correlates of general movements remain unclear. This analysis aimed to investigate the relationship between white matter integrity and general movements in infants with perinatal brain injury using advanced neuroimaging techniques. Methods: Diffusion-weighted MRI data were analyzed in 17 infants, 12 with perinatal brain injury and 5 typically developing infants. Tractography was used to identify the corticospinal tract, a key motor pathway often affected by perinatal brain injury, and tract-based spatial statistics (TBSS) were used to examine broader white matter networks. Diffusion parameters from the diffusion tensor imaging (DTI) and neurite orientation dispersion and density imaging (NODDI) models were compared between infants with and without typical general movements. Results: Corticospinal tract integrity did not differ between groups when averaged across hemispheres. However, infants with asymmetric general movements exhibited greater corticospinal tract asymmetries. A subset of infants with atypical general movement trajectories at <6 weeks and 3–5 months of age showed reduced corticospinal tract integrity compared to those with typical general movements. TBSS revealed significant differences in white matter integrity between infants with typical and atypical general movements in several white matter pathways, including the corpus callosum, the right posterior corona radiata, bilateral posterior thalamic radiations, the left fornix/stria terminalis, and bilateral tapetum. Conclusions: These findings support and expand upon previous research suggesting that white matter integrity across multiple brain regions plays a role in the formation of general movements. Corticospinal integrity alone was not strongly associated with general movements; interhemispheric and cortical-subcortical connectivity appear critical. These findings underscore the need for further research in larger, diverse populations to refine early biomarkers of neurodevelopmental impairment and guide targeted interventions.

## 1. Introduction

Perinatal brain injury (PBI), which occurs from early gestation through the first month after birth, frequently results in lifelong disabilities, including cerebral palsy (CP) [1]. Early identification of infants at risk for CP and timely access to rehabilitation intervention are critical to positively influence the developmental trajectory of at-risk infants and lower overall costs of care [2,3]. However, early and accurate biomarkers for prognosis are needed to further understand the neurodevelopmental pathways leading to CP.

The General Movements Assessment (GMA) is an evidence-based clinical tool with high sensitivity and specificity for predicting CP [4,5]. The GMA assesses spontaneous infant movement patterns, providing insights into motor system development with implications for development in non-motor domains as well [4,5]. The GMA evaluates movements in two infant age ranges: preterm or writhing movements before 8 weeks of age (corrected for prematurity, “Writhing age”), and Fidgety movements (FMs) between 9 and 20 weeks corrected age (“Fidgety age”), with optimal FM observation time between 12 and 16 weeks corrected age [6,7,8]. Absent FMs during this period have high specificity (96–98%) and sensitivity (95–100%) for CP diagnosis [6]. However, while the GMA has consistently demonstrated high predictive value for CP [4,5], the neurological correlates of general movements (GMs) are poorly understood [9]. A mechanistic understanding of how GMs relate to neurodevelopment could enhance prognostic accuracy, identify targets for individualized early intervention strategies, and lead to increased implementation of the GMA as an evidence-based clinical biomarker [9].

Neuroimaging studies have identified that absent FMs are associated with varied neurological findings including white matter abnormalities, reduced cerebellar transverse diameter, and basal ganglia and thalamic damage [10]. Functional networks have also been found to differ in preterm infants with typical and atypical GMs, demonstrating decreased connectivity between cortical regions integrating visual, somatosensory, and auditory information with deep gray matter nuclei [11]. Atypical GMs are associated not only with CP but are also observed in populations with Down syndrome, genetic syndromes, and autism spectrum disorders, and have been associated with cognitive, language, and other developmental delays [11,12,13,14,15].

Diffusion-weighted MRI (dMRI), a non-invasive method for assessing white matter integrity, has been used to investigate these structural correlates [16]. Diffusion tensor imaging (DTI) is the most commonly applied technique to model dMRI signals, allowing for the measurement of several parameters related to structural integrity and water diffusion [17]. Quantitative metrics of mean diffusivity (MD), the average diffusion across three directions, and fractional anisotropy (FA), the normalized standard deviation of the individual diffusivities, can be computed and used to describe the white matter microstructure. Additionally, while the diffusion tensor in DTI is a mathematically defined construct used to represent the dMRI signal, recent biophysical modeling techniques attempt to incorporate specific tissue microstructure features as model parameters whose effects are captured by the dMRI signal. One such technique is the neurite orientation dispersion and density imaging (NODDI) model, a biophysical diffusion model assuming diffusion in three compartments: intracellular, extracellular, and cerebrospinal fluid (CSF) [18]. Compartment-specific parameters estimated from NODDI have the potential to provide biologically relevant biomarkers specific to PBI, as these more explicitly attribute dMRI signal characteristics to features of the underlying biology [18]. These methods can identify subtle white matter differences that may not be visible with conventional neuroimaging.

Studies of infants with PBI suggest that dMRI can add to existing clinical assessments to identify infants at risk for atypical motor development, particularly when other imaging and motor assessment findings are unclear [19,20]. For example, measuring FA of the corticospinal tract (CST) offers added benefit over conventional neuroimaging in classifying [21] and predicting [20,22] future motor outcomes in infants with PBI. The CST is the primary voluntary motor output pathway of the brain and is commonly impacted by PBI. It undergoes substantial development over the first year of life as it is pruned from a bilateral to predominately contralateral circuitry, and as the motor cortex assumes an increasing role in motor outflow [23,24]. The extent to which the developing corticospinal integrity influences spontaneous movements is not well understood, though both CST integrity in three-month old infants [20,22] and GMs at the same age [6] are predictive of CP. Differences in white matter integrity in sensorimotor tracts also significantly correlate with motor function in later childhood in individuals with CP [25,26,27,28].

Brain pathways associated with typical and atypical GMs likely extend beyond the CST into both motor and non-motor regions [29]. TBSS is a DTI-based analytical method for the analysis of white matter microstructure which aims to combine the strengths of voxel-based analyses (not requiring pre-defined tracts or regions of interest) with the strengths of tractography analyses (limiting estimates to prominent white matter voxels) [30]. TBSS allows analysis of the connections between various brain regions without needing to specify tracts of interest a priori. Given the expansive re-organization that can occur after PBI, the variety of lesion types and locations, and the likelihood that GMs in infancy are associated with both motor and non-motor developmental outcomes, TBSS may be an appropriate tool to identify white matter regions of difference between infants with and without typical GMs.

This analysis aimed to identify the relationship between GMs and white matter integrity in infants with and without PBI. Specifically, we hypothesized that (1) infants with atypical GMs would demonstrate lower CST integrity compared to infants with typical GMs, (2) asymmetries in CST integrity would correlate with asymmetries in GMs and (3) as an exploratory aim, that widespread white matter pathways would show reduced integrity in infants with atypical GMs. By combining tractography and TBSS methodologies, this study provides new insights into the neural substrates underlying GMs and their potential role as biomarkers for early neurodevelopmental impairment.

## 2. Materials and Methods

### 2.1. Participants

Infants with PBI were recruited as part of a longitudinal observational study (NIH R01HD098202) according to a published protocol [31]. Infants were included if they had radiologically confirmed intraventricular hemorrhage (IVH), periventricular leukomalacia (PVL), hypoxic–ischemic encephalopathy (HIE), or acute brain lesions including neonatal hemorrhagic or thrombotic stroke. The exclusion criteria included (1) metabolic disorders; (2) chromosomal abnormalities or congenital syndromes; (3) neoplasm; (4) uncontrolled seizures or active seizure disorder on medications; (5) disorders of cellular migration and proliferation; (6) acquired traumatic brain injury; (7) surgeries that may constrain spontaneous movements; (8) other neurologic disorders unrelated to perinatal stroke/IVH/HIE/PVL; (9) mechanical ventilation, (10) indwelling metal or incompatible medical devices; and (11) ongoing apneic episodes and/or syncope.

A separate cohort of typically developing infants (NIH R00MH110596) was recruited as controls. These infants were born full-term (≥37 weeks gestational age). The exclusion criteria included (1) contraindications to MRI; (2) any neurological conditions; (3) known factors that may affect neurological or psychological development or health; (4) developmental, genetic, or metabolic disorders; (5) autoimmune disorders; (6) vascular or heart conditions; (7) cancer; (8) pre/postnatal drug exposure; (9) major surgery or invasive procedure; (10) low blood sugar; (11) metabolic disorder; (12) organ or structural malformation; and (13) major ultrasound abnormality.

Infants from the above studies were included in this analysis if they had a complete dMRI dataset obtained between the ages of term to 6 months 30 days. Infants with PBI were included if they had additionally completed a GMA in the Fidgety period (between 9 and 20 weeks of age). Infant ages were corrected for prematurity when applicable (for infants born < 37 weeks).

Informed consent was obtained from parents/guardians of all participants. Both studies were approved by the University of Wisconsin-Madison Institutional Review Board.

### 2.2. General Movements Assessment

For infants with PBI, the GMA was recorded via a 3- to 5-min video according to established protocols [32]. Videos were recorded either by the research team or by the infant’s parent/guardian at home with appropriate instructions. The infants were awake and calm, positioned supine, and without the use of a pacifier. Two raters, one of whom was blinded to diagnosis and neuroimaging, independently assessed and scored each GMA video. The final rating was determined by consensus. If the infants were unable to complete a Writhing (<8 weeks) and/or a Fidgety (9–20 weeks) GMA video as part of the research study (e.g., due to age at enrollment), GMA scores from a certified outside assessor (e.g., medical provider) were obtained from medical records when available.

For infants with absent FMs, raters additionally assessed whether asymmetries were observed, i.e., if FMs were observed on one side of the body but not the other. A motor optimality score (MOS) was computed by two raters at the Fidgety period, which includes GMs and other age-appropriate movement patterns, with a maximal (most optimal) score of 28 [10]. Optimality scores were averaged between raters for analysis. If the Fidgety GMA was obtained via medical record review (n = 1), infants were not included in analyses of the MOS or asymmetries.

### 2.3. MRI Acquisition

The infants were scanned during natural sleep on a 3 Tesla Discovery MR750 MRI scanner (GE Healthcare, Waukesha, WI, USA) with a 32-channel head coil. One infant was scanned on a 3 Tesla head-only high-performance gradient system (MAGNUS, GE Healthcare, Waukesha, WI, USA). Infants with PBI were scanned at up to two timepoints, the first between the ages of term to two months (Writhing age), and the second between the ages of three to six months (Fidgety age). Typically developing infants were scanned at one timepoint between the ages of one to six months.

T1-weighted imaging was obtained using either the GE MPRAGE sequence or a custom MPnRAGE sequence, a self-navigated imaging method that provides high-resolution motion corrected T1-weighted images and T1 relaxometry maps [33,34]. The MPRAGE sequence was acquired with 1 mm isotropic resolution, repetition time (TR) = 2.36 s, inversion time (TI) = 1.06 s, Echo Time (TE) = 0.004 s, Flip Angle = 8 degrees, slice thickness = 1 mm, and field of view (FOV) 160 × 256 × 256. The MPnRAGE scans were obtained with a 3D radial acquisition sequence using sinusoidal gradients. The parameters included 1 mm isotropic resolution, 160 axial slices, and a 256 × 256 in-plane acquisition matrix. The timing parameters were TR = 7.728 ms, TE = 2.3 ms, and TI = 2.3 ms. The inversion recovery curve was sampled with n = 256 views split at 192 with a 4-degree flip angle and 64 with an 8-degree flip angle. A composite T1-weighted image was obtained from all acquired n = 256 views and used in the analysis pipeline to confirm lesion locations and guide image registration.

A multiple b-value dMRI dataset was also acquired. The protocol included 99 diffusion-encoded volumes with b-values in s/mm^2^ of 300 (9 directions), 800 (15 directions), 1200 (30 directions), and 2000 (45 directions). Additionally, 6 volumes with no diffusion encoding (b = 0 s/mm^2^) were acquired. For two scans used in this analysis, the b = 300 s/mm^2^ volumes were not acquired due to an error in the scan protocol. An additional dMRI scan with 6 b = 0 s/mm^2^ volumes, 1 b = 300 s/mm^2^ volume, and 1 b = 2000 s/mm^2^ volume was acquired with reverse phase encoding to correct for susceptibility distortions [35]. Multiband imaging (factor = 2) was used to shorten the acquisition time. Additional scan details included in-plane resolution = 2 mm × 2 mm, acquisition matrix = 112 × 112 × 72, TE = 107 ms, Echo spacing = 0.902 ms, TR = 7000 ms, and flip angle = 90 degrees. Scan sequence parameters for the MAGNUS scan are included in Appendix A.

### 2.4. MRI Processing and Analysis

#### 2.4.1. Preprocessing

dMRI processing included the removal of Rician noise [36], removal of Gibbs ringing artifact [37] and correction for eddy-currents and motion using the FMRIB Software Library version 6.0.5 (FSL)’s EDDY tool [38] with outlier replacement [39] enabled. The *topup* routine from FSL was used alongside EDDY to estimate and correct susceptibility distortions [40]. One scan had significant motion artifact in the reverse phase-encoded direction leading to failure in using FSL’s EDDY and was processed via the TORTOISE DIFF PREP platform, which allows for incorporating a structural image for improved motion and distortion correction [41,42]. The diffusion tensors were calculated from pre-processed dMRI data using the Diffusion Imaging in Python software package version 1.9.0 (DIPY) [43]. The DTI parameters, including Fractional Anisotropy (FA), Mean Diffusivity (MD), Axial Diffusivity (AD), and Radial Diffusivity (RD), were computed using all diffusion shells [44]. The data were also processed according to the NODDI model [18] using the Diffusion Microstructure Imaging in Python (DMIPY) toolbox [45] as well as the Constrained Spherical Deconvolution (CSD) model, part of the MRtrix3 platform [46,47,48]. The response functions from the CSD model were computed using a multi-shell multi-tissue approach using the *dhollander* algorithm, which does not require prior tissue type segmentation [49]. Intracellular volume fraction (ICVF), orientation dispersion index (ODI), and isotropic volume fraction (ISOVF), were computed from the NODDI model, and white matter fiber orientation distribution function (fODF) was computed from the CSD model.

#### 2.4.2. Tractography

Based on the a priori hypotheses, TractSeg v2.9 was used to generate white matter bundle segmentations and probabilistic tractograms for the CST for all scans [50]. TractSeg makes use of a deep learning model trained on data aligned to MNI space [50,51]. Therefore, to improve tract segmentations, affine registration of the fODF maps to the MNI-152 T1 1 mm template [52] was performed using ANTS [53] prior to running TractSeg. TractSeg consists of a multi-step process based on the fODF map. This process starts with segmentation of a voxelwise probability (0,1) mask for a given tract, which is then binarized based on a default probability threshold value of 0.5. This is further classified as a beginning and end mask. Tract orientation maps are then estimated from the white matter fODFs for each tract of interest based on the binarized tract segmentations, and tract-specific probabilistic fiber tracking is performed using the tract orientation maps and beginning and end masks. If white matter bundle segmentations were identified for a given tract but tractograms were not produced, TractSeg was re-run with an alternate thresholding. The threshold for converting the tract probability maps to binary maps was set at 0.05 to include a greater number of putative tract voxels in binary maps. Then, to ensure that only the largest contiguous voxel cluster representing the tract of interest was included, clusters of less than 500 voxels were removed. All tractograms were visually inspected for quality control. Once the tractograms were obtained, tract density images were computed and thresholded to remove voxels with high CSF content (ISOVF > 0.5) or voxels with a value of <5% of the robust maximum (98th percentile) of the streamline distribution. The parameter values were calculated as weighted averages across the tract by dividing the mean of the tract by the robust maximum of the streamline probability distribution.

#### 2.4.3. TBSS

To investigate additional brain regions that may be associated with GMs, an exploratory TBSS analysis was performed. First, a study-specific template was created from the five typically developing infant scans using the ANTS BuildTemplateParallel.sh script (ANTS version 2.3.3), using affine registration first followed by diffeomorphic registration with the Greedy Symmetric Normalization (SyN) algorithm [53]. FA maps from each participant were non-linearly warped to the study-specific template space for analysis using FSL FNIRT [40]. The resulting warp fields were then applied to the other DTI and NODDI parameter maps. For participants with cortical lesions or substantial ventricular dilation, a lesion mask was used to guide registrations. Lesion masks were created from the T1 structural image using a combination of manual and automated tools in ITK-SNAP [54], and registered to diffusion space using rigid transforms with FSL FLIRT [40,55,56]. All registrations were visually inspected for accuracy.

Voxelwise statistical analysis was performed using TBSS [29,30]. Infants were divided into two groups, namely (1) typically developing infants and infants with PBI and normal FMs and (2) infants with PBI with absent FMs. For participants with PBI, the scan obtained at Fidgety age was used, with two exceptions. The Writhing age scans for two participants (the participant scanned on the MAGNUS system at Fidgety age [group: Normal FMs] and the participant processed with TORTOISE due to excessive motion at Fidgety age [group: Absent FMs]) were used in the TBSS analysis to minimize variability in the sample due to scan acquisition or image processing differences. Once registration was achieved for all participants, a mean FA image was created and thinned to create a mean FA skeleton, representing the center of tracts common to the population. A threshold of 0.2 was applied to generate the skeleton. After alignment to the study-specific template, participant parameter maps were projected onto the white matter skeleton for voxelwise statistical analysis.

### 2.5. Statistical Analysis

#### 2.5.1. Tractography

Statistical analyses of CST tractography were completed in R version 4.3. The scan closest to the age of the fidgety GMA was used for all participants in GLM and asymmetry analyses. Tract parameters from the DTI (FA, MD, RD, AD) and NODDI (ICVF, ODI, ISOVF) models were compared between groups ((1) typically developing infants and infants with PBI and normal FMs and (2) infants with PBI with absent FMs) with a general linear model (GLM) to control for sex and age at time of scanning. The mean of the weighted average parameter values for the CST was computed between hemispheres. Participants with only a unilateral CST were excluded from this analysis. Weighted average parameter values were also compared to motor optimality scores via a GLM.

Hemispheric asymmetry indices (AI) were calculated for each metric using the equation: AI = (more affected − less affected)/(more affected + less affected) for infants with unilateral brain injury, or AI = (left − right)/(left + right) for TD infants or infants with bilateral injury. Infants with only a unilateral CST were excluded from this analysis. Asymmetry indices were compared among infants with asymmetries noted on GMA (e.g., FMs on one side of the body and not the other) and those without asymmetries using Mann–Whitney U tests.

For all tractography analyses, *p*-values were compared at a level of 0.05. A Bonferroni correction was also calculated to account for multiple comparisons (e.g., across the seven diffusion metrics assessed), with a corrected a level of 0.007.

An exploratory analysis was conducted for the 5 participants with scans at Writhing and Fidgety age to investigate trends in FA over time among participants with different trajectories of GMs.

#### 2.5.2. TBSS

For TBSS, a general linear model was used to investigate group-wise white matter microstructural differences, controlling for sex and age at time of scanning using FSL *randomise*. Analyses were corrected using the Family-Wise Error Rate for multiple comparisons by using threshold-free cluster enhancement [57]. Due to the small sample size and corrections for multiple comparisons, *p*-values were compared to two different alpha levels for determining significance, α = 0.05 and α = 0.1. The JHU ICBM-DTI-81 white matter labels atlas was registered via ANTS diffeomorphic registration to the mean FA image generated by the TBSS pipeline for identification of significant white matter regions. Atlas regions containing > 5 significant voxels from the FA skeleton were considered significant.

## 3. Results

**Figure 1:**
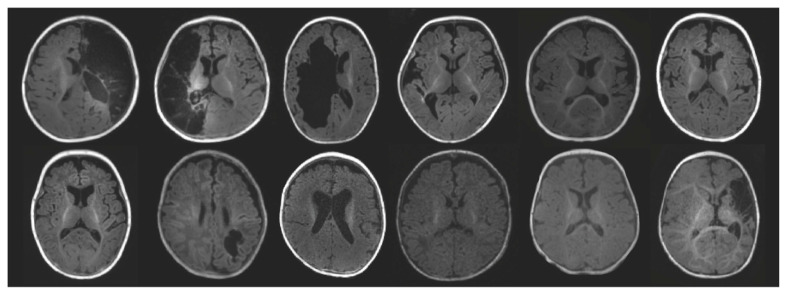
Structural T1 images for participants with perinatal brain injury (n = 12). The figure contains a single axial slice depicting the lesion (when applicable) for each participant.

Twelve infants with PBI and five typically developing infants met inclusion criteria for this analysis. Seven participants in the PBI group had Absent FMs on the GMA. Demographic and radiological summary information about the infants is included in Table 1. T1 images from infants with PBI demonstrating lesion location and extent are depicted in Figure 1. All infants with PBI had a scan and GMA completed at the Fidgety age range. Five infants with PBI had imaging and GMA completed at both Writhing and Fidgety age ranges. The five typically developing infants had one scan completed between the ages of 1–6 months.

### 3.1. Tractography

**Figure 2:**
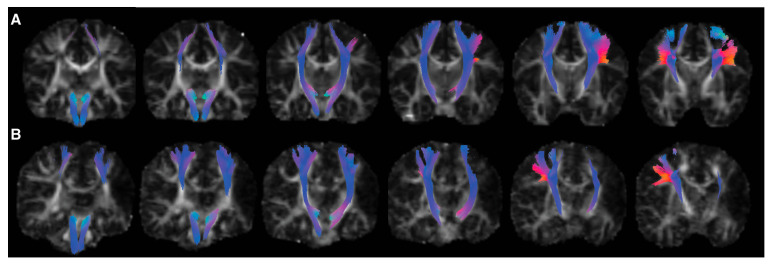
Example corticospinal tracts generated by TractSeg in (**A**) a typically developing participant and (**B**) a participant with a left-hemispheric perinatal stroke. Images are in radiological display convention. The tract color represents orientation. Six coronal slices are depicted from posterior to anterior, with tractograms overlaid on top of fractional anisotropy images. Tractograms and FA images were aligned to MNI space using affine transformation. Decreased branching was noted in the corticospinal tract of the lesioned hemisphere of the participant with perinatal stroke (**B**).

**Figure 3:**
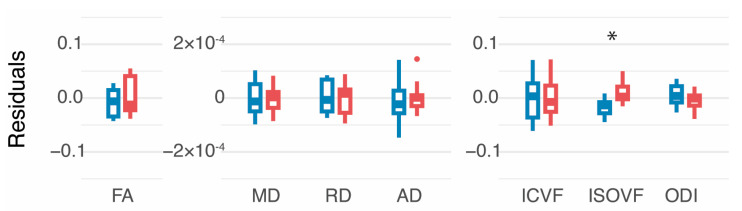
White matter integrity in corticospinal tracts, averaged across hemispheres. For each parameter, the relationship with age and sex was modeled with a general linear model. Boxplots depict the residuals for each participant by group. Infants with present fidgety movements are shown in blue (left) and infants with absent fidgety movements are shown in red (right). FA = fractional anisotropy, MD = mean diffusivity, RD = radial diffusivity, AD = axial diffusivity, ICVF = intracellular volume fraction, ISOVF = isotropic volume fraction, ODI = orientation dispersion index. * significant at *p* = 0.05.

Tractograms were generated for all participants. Two participants with PBI did not have a CST identified in the lesioned hemisphere. Figure 2 shows example TractSeg output for the CST for a typically developing infant and an infant with PBI. No DTI or NODDI parameters of the CST showed significant differences based on GMA result (absent vs. present FMs) at the corrected α level. There was a significant difference in ISOVF compared to the uncorrected α level of 0.05 (*p* = 0.03) after adjusting for age and sex. There was a significant relationship between MOS and FA (*p* = 0.03) after adjusting for age and sex when compared to the uncorrected α level of 0.05 (Supplementary Figure S1). There were no other significant relationships between MOS and tract parameters. Figure 3 plots the residuals by group (normal vs. absent FMs) obtained from GLMs of the effect of age and sex on DTI and NODDI tract parameters. In a subsample with unilateral injuries, no significant differences by group in any tract parameters were seen in lesioned or non-lesioned hemisphere CSTs (Supplementary Table S1).

**Figure 4:**
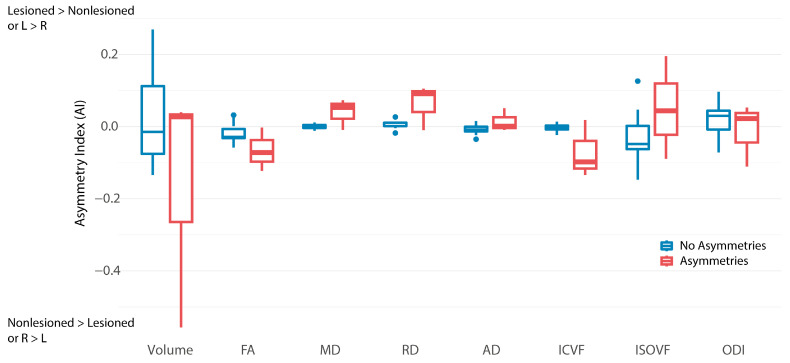
Tract asymmetry indices for the corticospinal tract for infants without (blue, left) and with (red, right) asymmetries noted on the General Movements Assessment (GMA). Two children with asymmetries on the GMA did not have a lesioned hemisphere corticospinal tract and are not included in this plot. FA = fractional anisotropy, MD = mean diffusivity, RD = radial diffusivity, AD = axial diffusivity, ICVF = intracellular volume fraction, ISOVF = isotropic volume, ODI = orientation dispersion index, R = Right, L = Left.

Five participants in the sample had absent FMs with asymmetries, three of whom had bilateral CSTs allowing calculation of AIs. The CST AIs for all parameters were not significantly different between infants with asymmetries noted on the GMA and those without asymmetries; however, infants with asymmetries on the GMA tended to have a wider range of AIs, and mean AIs further from zero (Figure 4).

**Figure 5:**
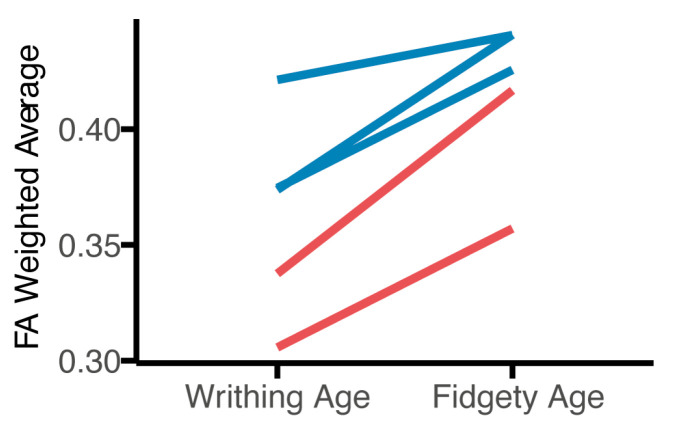
The exploratory longitudinal findings (n = 5) for fractional anisotropy (FA) weighted average. The mean FA weighted average was taken between both hemispheres. All infants had poor-repertoire General Movements Assessment at Writhing age. Blue lines represent infants that developed normal fidgety movements; red lines represent infants that developed absent fidgety movements.

Exploratory longitudinal comparisons of FA were conducted for participants with both a Writhing and Fidgety age MRI scan and GMA (n = 5). Figure 5 depicts changes in FA of evaluated tracts over time. All five participants had poor repertoire GMs on their Writhing GMA. Notably, the two participants who went on to develop absent FMs had lower FA at both the Writhing and Fidgety timepoints in the CST compared to the three participants who developed normal FMs.

### 3.2. Tract-Based Spatial Statistics

**Figure 6:**
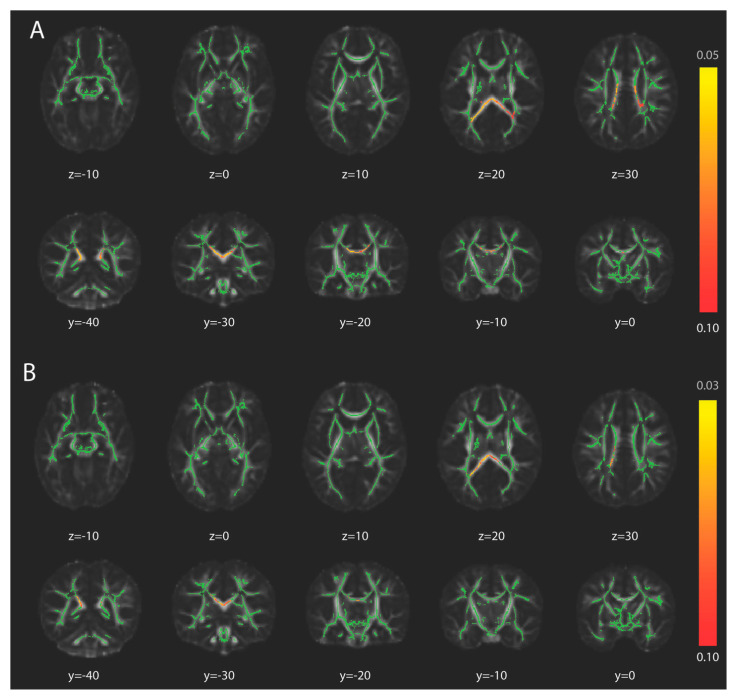
White matter differences between infants with normal and absent fidgety movements. The fractional anisotropy (FA) skeleton, in green, is overlaid on top of the study-specific FA template, aligned to MNI space. (**A**) The colored voxels on a yellow-red color scale indicate significantly higher FA in infants with normal fidgety movements compared to infants with absent fidgety movements. (**B**) The colored voxels on a yellow-red color scale indicate significantly lower orientation dispersion index in infants with normal fidgety movements compared to infants with absent fidgety movements. Z represents the MR imaging axial coordinates and Y represents the MR imaging coronal coordinates based on the MNI template. The color map indicates *p*-value.

White matter tracts with voxels with significantly lower FA in infants with absent FMs at *p* < 0.1 included the body and splenium of the corpus callosum, the right posterior corona radiata, the right and left posterior thalamic radiations (including optic radiations), and the right and left tapetum (Figure 2). There were no tracts with significant differences in FA at *p* < 0.05. White matter tracts with voxels with significantly higher ODI in infants with absent FMs included the body and splenium of the corpus callosum at *p* < 0.05. At *p* < 0.1, ODI was also significantly higher in infants with absent FMs in the left posterior thalamic radiation and left tapetum (Figure 6). There were no tracts with significantly higher FA or significantly lower ODI in infants with absent FMs. There were no tracts with significant differences in MD, AD, RD, ISOVF, or ICVF at either α level.

## 4. Discussion

This study investigated the relationship between white matter integrity and GMs in infants with PBI and typically developing infants. Our findings provide evidence that multiple white matter pathways, particularly interhemispheric and cortical-subcortical connectivity, are associated with typical GMs. There were not significant differences in CST integrity between infants with and without typical GMs when averaged across hemispheres. However, infants with asymmetric spontaneous movements tended to exhibit greater CST asymmetry, suggesting that early motor asymmetries may reflect underlying microstructural differences in motor pathways.

Among our samples, we did not find overall differences in CST integrity between infants with normal and absent FMs with the TractSeg analysis. Previous research investigating CST integrity as a predictor for motor development and CP included infants with unilateral brain injury and focused on CST integrity in the affected hemisphere [20,22]. However, less is known about the relationship between bilateral injury, CST integrity at 0–6 months, and motor outcomes. Additionally, while the CST is a primary motor output pathway in children and adults, current evidence suggests that it is not a key driver of movement in young infants [23]. Transcranial magnetic stimulation studies have shown that CSTs are functionally intact early in development, with the ability to elicit a motor response by activating the motor cortex [58,59]. However, current evidence from mammalian animal models indicates that shortly after birth, the motor cortex acts mainly as a sensory structure, likely assuming a primary role in motor outflow over the first year of life [23]. The developmental timeline over which CSTs become a primary contributor to motor output remains poorly understood.

We also investigated asymmetries in motor pathway integrity in infants with higher probability of developing unilateral CP (e.g., those with absent FMs on one side of the body). At birth, CSTs are organized bilaterally, descending from each hemisphere to bilateral upper extremities. At around 3–5 months of age, ipsilateral CSTs begin to undergo pruning, such that remaining hemispheric control of each limb is primarily contralateral [24]. However, after damage due to unilateral or asymmetrical perinatal stroke, the ipsilateral pathways from the non-lesioned hemisphere are sometimes strengthened, while the crossed CST from the lesioned hemisphere may be absent or weakened [60]. This has been demonstrated in adolescents with unilateral CP, where asymmetries were found in white matter integrity of the CSTs [61]. In the present investigation, in two participants with asymmetric FMs, the lesioned-hemisphere CST was unable to be reconstructed, likely due to lesion location and size. Although not statistically significant, we noted that infants with bilateral tracts and asymmetric FMs displayed a trend toward lower white matter integrity in lesioned-hemisphere tracts. This may indicate the early emergence of asymmetries in both corticospinal integrity and movement that are frequently associated with unilateral CP. Corticospinal asymmetries may continue to emerge as these infants develop. Asymmetries in GMs at this age may also reflect asymmetries in other brain regions or in interhemispheric excitation/inhibition imbalance.

In brain regions that demonstrated significant differences on TBSS analysis, white matter integrity was lower in infants with absent FMs than in infants with normal FMs and typically developing infants, as indicated by lower FA values. Furthermore, ODI values were significantly higher in many of the same regions in infants with absent FMs, but there were no significant differences in ICVF, suggesting that a reason for the lower tract integrity may be greater dispersion rather than lower neurite density. Previous work has suggested that the combination of higher ODI and lower FA is indicative of lower organization in white matter regions [62]. While the relationship between PBI and ODI is poorly understood, greater ODI has been found throughout white matter in children born very preterm compared to children born at term, and greater ODI in various white matter tracts has been correlated with poorer neurodevelopmental outcomes in very preterm children [62,63]. Three participants in this sample were born at <37 weeks (one with normal FMs, two with absent FMs). Future work should further evaluate differences in ODI between preterm and term-born infants with PBI.

A previous study by Peyton et al. (2017) incorporated TBSS in an analysis of differences in white matter integrity between infants with and without typical GMs [29]. This study included a population of very preterm infants who were scanned at term, 38 of whom had normal FMs and 9 of whom had abnormal FMs. Peyton et al. found lower FA in the corpus callosum, inferior longitudinal and fronto-occipital fasciculi, internal capsule, and optic radiation [29]. Another recent study by Tabacaru et al. (2024) used TBSS to perform a similar analysis in infants with HIE with and without typical GMs [64]. This study included 17 infants with normal FMs and 3 infants with absent FMs and found decreased FA in the posterior limb of the internal capsule, the cingulum, the corpus callosum, and posterior thalamic/optic radiations. Many of the regions identified in the present work are similar to the findings in these two prior studies; the differences could be explained by the older age of infants at scanning, inclusion of both preterm and term-born infants, the more extensive and heterogeneous pathologies included in the present investigation, and/or the smaller sample in the present investigation compared to the Peyton et al. study.

Previous neuroimaging studies have associated atypical GMs with varied neurological findings including in the cerebellum, sensorimotor cortex, corpus callosum, internal capsule, basal ganglia, and thalamus, as well as widespread white matter abnormalities [10,29,65]. Hadders-Algra (2018) proposed that the key neural driver for impaired GMs is impaired connectivity in widespread cortical-subcortical networks [66]. In the current analysis, brain pathways that were significant in TBSS are related to interhemispheric and cortical-subcortical connectivity, supporting this theory. Hadders-Algra suggests that the cortical subplate and the connecting white matter are dominant drivers of GMs particularly in the preterm/writhing ages, and the transition from writhing to FMs occurs as motor control is gradually taken over by the cortical plate [66]. The cortical subplate is a transient structure that receives input from the thalamus and projects to the developing cortex and is particularly susceptible to injury in the perinatal period [67]. It is likely that there are many structural and functional networks involved in the generation of spontaneous infant movements, which helps explain why atypical GMs are associated not only with later movement impairment but also cognitive, language, and other developmental delays [11].

Limitations to this investigation include the number of infants analyzed, the varied pathologies represented in the study, and methodological challenges with performing imaging analysis with an infant sample with atypical brain morphology. Due to the small sample size, we may have been underpowered to detect all differences in white matter tract integrity that may be associated with atypical GMs. Despite our limited sample, the agreement between many of the brain regions identified via TBSS analysis in this study and other studies in similar populations [29,64] supports the likely contribution of the identified white matter pathways to GMs. The disparate lesion locations and types may have also impacted our analyses. In particular, it is not currently well understood how NODDI parameters may be impacted by different types of PBI. While this investigation expands upon previous work by including infants with multiple injury mechanisms, future work in larger sample sizes should aim to further stratify analyses by injury type to control for this potential source of heterogeneity. A future study with three groups (typically developing infants, infants with PBI and absent FMs, and infants with PBI and normal FMs) would further strengthen our ability to elucidate specific changes related to atypical GMs compared to the brain injury itself. A GMA video was not acquired for typically developing infants who were assumed to have normal FMs for this analysis. Due to the strict inclusion criteria, it is unlikely that infants in the typically developing sample had neurological risk factors that may be associated with absent FMs. Furthermore, no brain pathology was identified after radiological review of the typically developing infant MRI scans.

The analysis methods employed in this investigation were designed and tested with adult datasets and required specific modifications to be successfully implemented in infants. For example, TractSeg was trained on the Human Connectome Project dataset. As a component of the analysis pipeline, TractSeg produces tract beginning and ending binary masks as well as tract bundles that are guided by adult neuroanatomy. In some cases, the beginning or ending mask for a given tract was not able to be constructed, yielding an empty bundle segmentation. This does not account for possible neuroplasticity and relocation of the cortical region representing the beginning or ending of a particular tract, leaving the possibility that TractSeg did not identify tracts for some infants in which they were present; however, this occurred only in two infants with large lesions in the hemisphere with the missing tract. Recent work utilizing TractSeg for adults with hemispherectomy similarly identified the possibilities of false negative results due to non-overlapping bundle and beginning/ending masks as well as false positives in lesioned areas [68]. The authors emphasized the need for manual confirmation of segmentation quality in populations with large brain pathologies, which was incorporated into our methodology [68]. TractSeg has previously been successfully used in pediatric populations, including a large population of infants born very prematurely [69] and adult populations with brain pathologies [70]; however, future validation of this tool for infants with brain injury is needed. Future analyses incorporating tractometry could identify local tract differences along segments of the tracts that may have been masked by methodologies employing whole-tract weighted averaging. Additionally, TBSS requires registration of individual participant images to a standardized template. Some of the infants experienced anatomical impacts such as ventricular dilation or hemispheric shift in association with their brain injury, and registration may have required more extensive non-linear image warping for infants with these anatomical differences than infants with more typical neuroanatomy. A study-specific template created from the typically developing infants in the sample helped improve registration quality compared to using an adult or single-subject target image. However, the study-specific template did not include infants with PBI due to limitations in the methodology for template creation (e.g., no option to include lesion masks in the registration algorithm), which may have resulted in higher registration inaccuracy in the infants with PBI. A strength of TBSS analysis is that it is relatively robust to lesions, small amounts of motion, and anatomical variations because the white matter skeleton represents the centers of the tracts, which increases the validity of the TBSS analysis method for this population of infants with brain lesions [30]. Continued development of infant-specific neuroimaging analysis tools and tractography tools that are robust to diverse neuroanatomy and pathology is needed.

Our analysis was limited to cortical and subcortical white matter. Brainstem pathways, which may be highly involved in the formation of early infant movements, did not show differences with TBSS in this sample, which could be attributed to the limited resolution of the methods employed to assess brainstem circuits. Future studies may also consider investigating differences in subcortical gray matter regions or in functional connectivity patterns that may relate to GMs, which the present analysis did not explore. As one example, a past study used resting-state functional MRI and identified atypical connectivity between the basal ganglia and regions in parietal and frontotemporal lobes in infants with atypical GMs compared to infants with typical GMs [11]. Future work implementing techniques like functional near-infrared spectroscopy or magnetoencephalography may additionally allow for real-time assessment of cortical and subcortical function occurring while infants are awake and engaging in spontaneous movement.

## 5. Conclusions

This investigation aimed to identify associations between patterns of GMs and white matter connectivity in infants with and without PBI. The results highlight the likely importance of widespread white-matter connectivity, particularly interhemispheric and cortical-subcortical pathways, in the development of GMs in early infancy, and support previous literature in similar populations. While CST integrity alone was not strongly associated with GM outcomes in this sample, asymmetries in CST integrity and differences in other white matter regions were linked to atypical movement patterns. These findings advance our understanding of the neural substrates of GMs and emphasize the need for multimodal approaches in early developmental assessment. Future research with larger, longitudinal datasets and advanced imaging techniques will further elucidate the complex interplay between neural connectivity and early motor development.

## Figures and Tables

**Table 1:** Participant Characteristics.

Infants with Perinatal Brain Injury (N = 12)	
Sex	3F, 9M
Primary Diagnosis	
Ischemic or hemorrhagic perinatal stroke	6
Intraventricular hemorrhage	2
PVL	1
HIE	3
Lesioned Hemisphere	
Left	4
Right	4
Bilateral	4
Preterm/Writhing GMA (N = 5)	
Age at Writhing scan, weeks (mean (range))	7.4 (5.4–8.4)
Age at GMA, weeks post-term (mean (range)) *	2.8 (−3.7, 5.7)
Normal	0
Poor Repertoire	5
Cramped-Synchronized	0
Chaotic	0
Fidgety GMA (N = 12)	
Age at Fidgety scan, weeks (mean (range))	19.2 (13.4–29.4)
Age at GMA, weeks post-term (mean (range)) *	15.5 (12.2–18.1)
Normal Fidgety	4
Absent Fidgety	8
Abnormal Fidgety	0
Motor Optimality Score (median, range)	(9.5, 7–24)
**Typically Developing Infants (N = 5)**	
Sex	1F, 4M
Age at scan–weeks (mean (range))	13.7 (6.7–17.8)

GMA = General Movements Assessment, PVL = periventricular leukomalacia, HIE = hypoxic–ischemic encephalopathy, F = Female, M = Male. Ages are corrected for prematurity when applicable. * Ages at GMA are presented as weeks post-term regardless of infant gestational age, according to standard practice for this assessment.

## Data Availability

The datasets presented in this article are not readily available because some of the data are part of an ongoing study. Requests to access the datasets should be directed to the corresponding author.

## Supplementary Materials

The following supporting information can be downloaded at https://www.mdpi.com/article/10.3390/brainsci15040341/s1: Figure S1: Motor Optimality Score and Average Fractional Anisotropy; Table S1: Regression Analyses by Hemisphere.

## Appendix A

MAGNUS Scan Sequence (MPRAGE): 1 mm isotropic resolution, repetition time (TR) = 2.29 s, inversion time (TI) = 1.06 s, Echo Time (TE) = 0.004 s, Flip Angle = eight degrees, slice thickness = 1 mm, field of view (FOV) 160 × 256 × 256.

MAGNUS Scan Sequence (dMRI): 99 diffusion-encoded volumes were collected with b-values in s/mm^2^ of 300 (9 directions), 800 (15 directions), 1200 (30 directions), and 2000 (45 directions). In total, seven volumes with no diffusion encoding (b = 0 s/mm^2^) were acquired. An additional dMRI scan with six b = 0 volumes, one b = 300 volume, and one b = 2000 volume was acquired with reverse phase encoding. Multiband imaging (factor = 2) was used to shorten the acquisition time. Slice thickness = 2 mm, FOV = 112 × 112 × 74, TE = 113 ms, Echo spacing = 1.06 ms, TR = 7100 ms, Flip Angle = 90 degrees.
