# Supplementary material for: White-Matter Connectivity and General Movements in Infants with Perinatal Brain Injury
Source: Brain Sci. 2025 Mar 26;15(4):341. doi: 10.3390/brainsci15040341 (PMC12025426; doi:10.3390/brainsci15040341)
Supplement: Supplementary file 1 [file brainsci-15-00341-s001.zip › brainsci-3482064-supplementary.pdf]

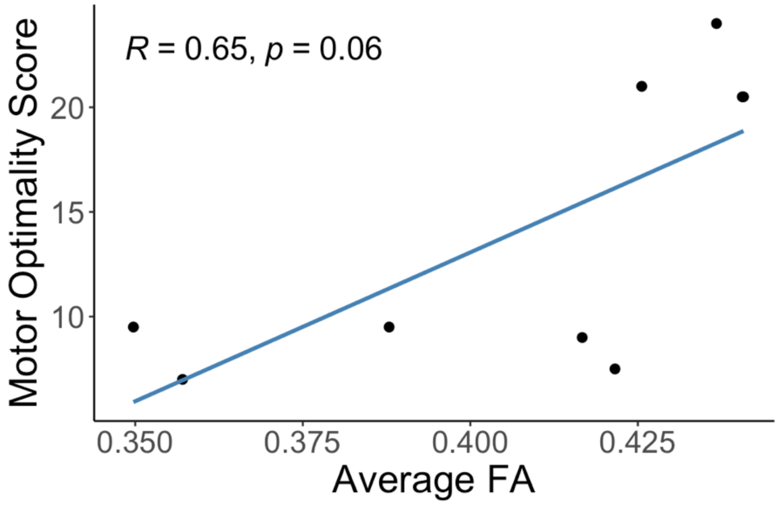

Supplemental Figure S1. Motor Optimality Score and Average Fractional Anisotropy. Scatter plot demonstrating the relationship between the Motor Optimality Score (MOS) and the weighted fractional anisotropy (FA) values averaged across both corticospinal tracts for infants with perinatal brain injury (n=8). The relationship approached significance with a Spearman correlation (p = 0.06, R = 0.65). After adjusting for age and sex, the MOS was significantly related to weighted average FA (p = 0.029).

Supplemental Table S1: Regression Analyses by Hemisphere. Regression analyses of diffusion metrics in lesioned and non-lesioned hemispheres in infants with normal vs absent fidgety movements

**Lesioned Hemisphere (n=6)**			
**Metric**	**Estimate**	**Standard Error**	**P-value**
FA	0.079	0.021	0.13
MD	.00000029	.000098	1.0
AD	0.00011	0.00010	0.41
RD	0.000055	0.000097	0.63
ICVF	0.0075	0.063	0.916
ODI	-0.043	0.020	0.16
ISOVF	0.036	0.019	0.20
**Non-lesioned Hemisphere (n=8)**			
**Metric**	**Estimate**	**Standard Error**	**P-value**
FA	-0.024	0.021	0.31
MD	0.000075	0.000053	0.23
AD	0.000084	0.000082	0.37
RD	0.000071	0.000043	0.18
ICVF	-0.054	0.038	0.22
ODI	-0.017	0.022	0.49
ISOVF	0.045	0.019	0.07

FA = Fractional Anisotropy, MD = Mean Diffusivity, AD = Axial Diffusivity, RD = Radial Diffusivity, ICVF = Intracellular Volume Fraction, ODI = Orientation Dispersion Index, ISOVF = Isotropic Volume Fraction. Estimates reflect the amount change in the metric based on the regression model for “normal fidgety” compared to “absent fidgety”. Analyses are adjusted for infant age and sex.
